# Choking as Cause of Death Among the Mentally Ill: A Literature review

**DOI:** 10.1192/j.eurpsy.2024.427

**Published:** 2024-08-27

**Authors:** P. Papadopoulou, G. N. Porfyri

**Affiliations:** Psychiatry Department, General Hospital of Papanikolaou, Thessaloniki, Greece

## Abstract

**Introduction:**

According to data, choking is one of the principal causes of death in mental health units. Specifically, research reveals that psychiatric patients -compared to the general population- are 43 times more likely to die due to choking. Nevertheless, only a limited number of studies has been focused on the risk factors of choking among this category of patients. Interestingly, dysphagia and choking on food are underdiagnosed and underreported in the UK psychiatry departments while there is an important insufficiency of provided information in national guidance archives as well as in regional clinical settings for adults with mental health diseases.

**Objectives:**

To explore the risk factors of choking among psychiatric patients and to highlight interventions of preventing choking-related incidents.

**Methods:**

A review of 36 articles -from 2010 to 2023- on PubMed and Google Scholar regarding choking-related incidents among inpatients of mental health units.Articles exploring choking suicide-related incidents or choking as cause of neurological illness, were excluded from the research. Keywords: choking, psychiatric patients, death.

**Results:**

Numerous risk factors of choking have been identified, such as:

Antipsychotics,

Anxiolytics,

Bradykinetic dysphagia (extra-pyramidal syndrome),

Intellectual disabilities,

Dementia,

Anxiety,

Coughing while eating,

Fast paced eating and cramming food,

Mealtime-related stressors such as willingness of avoiding peers.

**Conclusions:**

There is an absolute need for a specialized training of nurses, caregivers, mental health clinicians to prevent incidents and injuries of inpatients due to choking. Close supervision, routine screening during the mealtime, and detailed information from relatives about the patient’s eating habits are essential for the safety and the ameliorated quality of hospitalization for the mentally ill.

**Disclosure of Interest:**

None Declared

